# Determination of Intraprostatic and Intratesticular Androgens

**DOI:** 10.3390/ijms22010466

**Published:** 2021-01-05

**Authors:** Markéta Šimková, Jiří Heráček, Pavel Drašar, Richard Hampl

**Affiliations:** 1 Institute of Endocrinology, Narodni 8, 11694 Prague, Czech Republic; rhampl@endo.cz; 2 Department of Chemistry of Natural Compounds, University of Chemistry and Technology, Technicka 6, 16628 Prague, Czech Republic; Pavel.Drasar@vscht.cz; 3 Department of Urology, First Faculty of Medicine, Charles University, 12108 Prague, Czech Republic; Jiri.Heracek@lf1.cuni.cz; 4 Department of Urology, Military University Hospital, 16902 Prague, Czech Republic

**Keywords:** prostate, testes, cancer, determination, androgens, biomarkers, disease prediction, methods

## Abstract

Androgens represent the main hormones responsible for maintaining hormonal balance and function in the prostate and testis. As they are involved in prostate and testicular carcinogenesis, more detailed information of their active concentration at the site of action is required. Since the introduction of the term intracrinology as the local formation of active steroid hormones from inactive precursors of the adrenal gland, mainly dehydroepiandrosterone (DHEA) and DHEA-S, it is evident that blood circulating levels of sex steroid hormones need not reflect their actual concentrations in the tissue. Here, we review and critically evaluate available methods for the analysis of human intraprostatic and intratesticular steroid concentrations. Since analytical approaches have much in common in both tissues, we discuss them together. Preanalytical steps, including various techniques for separation of the analytes, are compared, followed by the end-point measurement. Advantages and disadvantages of chromatography-mass spectrometry (LC-MS, GC-MS), immunoanalytical methods (IA), and hybrid (LC-IA) are discussed. Finally, the clinical information value of the determined steroid hormones is evaluated concerning differentiating between patients with cancer or benign hyperplasia and between patients with different degrees of infertility. Adrenal-derived 11-oxygenated androgens are mentioned as perspective prognostic markers for these purposes.

## 1. Introduction

The testis is the major source of androgens as well as estrogens in men, while the prostate is one of the main sex steroid targets.

### 1.1. Prostate

#### 1.1.1. Intraprostatic Androgens

Intraprostatic steroid determination depends on the way and site of sample removal and its procession before end-point determination. While earlier studies required relatively high amounts of tissue (of the order of tens milligrams), recent more sensitive analytical methods such as various combinations of chromatographic techniques with mass spectrometry (MS) need less than 5 mg of tissue, as obtained by needle biopsy. The specificity of the methods and better separation of steroids enable to reduce of the pre-purification steps. In all instances, the immediate deep freezing of the tissue in liquid nitrogen and its storage at −70 °C is crucial since intraprostatic steroids undergo rapid metabolism. Early reports demonstrated that T in the prostate, after releasing from the androgen-binding protein (ABP), is reduced to its main saturated metabolite, dihydrotestosterone (DHT). DHT, compared to testosterone (T), has a higher affinity for the androgen receptor [1].

#### 1.1.2. Prostate Cancer and Androgen Deprivation Therapy

The importance of sex steroids actions for prostate physiology and pathology is emphasized concerning prostate cancer (PCa), which is one of the most frequent neoplasia in men. Androgens (T and DHT) and their metabolites play important roles in the disease. Through androgen/androgen receptor signaling, they are essential for development and function, as well as the proliferation and survival of epithelial cells within the prostate gland. Androgen deprivation therapy (ADT), either chemical, based on affecting hormone biosynthesis and androgen receptor activation or by surgical castration, has been used for decades to inhibit androgen-dependent PCa cell growth. Unfortunately, many patients will later develop hormone-resistant or castration-resistant cancer. On the other hand, despite the very low serum androgen levels, most castration-resistant cancers remain dependent on the androgen receptor signaling pathway. From this point of view, the intratumoral generation of DHT from adrenal androgen precursors, dehydroepiandrosterone (DHEA) and its sulfate and androstenedione, called intracrinology, is of importance [2].

In primates and humans, the active steroid hormones can be formed from inactive precursors of the adrenal gland, mainly DHEA and DHEA-S, where the local formation of active androgens depends on the expression of solute carrier organic anion transporter polypeptides (SLCO, formerly organic anion-transporting peptides (OATP)) and several androgen biosynthetic enzymes—sulfatase, 3β -hydroxysteroid dehydrogenases, 17β -hydroxysteroid dehydrogenases, and 5α -reductases—but also androgen metabolizing enzymes, sulfotransferases, and uridine 5′-diphospho-glucuronosyltransferase. Progression of castration-resistant PCa is accompanied by increased expression of steroid-5α-reductase. This enzyme exists in three different isoforms. Both SRD5A1 and SRD5A2 are expressed in the liver, where the latter is highly expressed in the androgen-sensitive tissue such as the prostate and testes. SRD5A2 is associated with microsomal membranes and facilitates the conversion of T to DHT, which is essential for the development of the normal male accessory sex organs [3,4]. Currently, there is limited evidence that SRD5A3 makes any contribution to the 5α-reduction of androgen in vivo, but rather, it seems to play a role in N-linked protein glycosylation [5].

As demonstrated by in vitro experiments with fresh prostatic tissue, the concentration of DHT decreases by more than half within 2 h of incubation at 37 °C, indicating the further metabolism of DHT. The saturated 5α-androstanediols, either free or conjugated with glucuronic acid, are the main products [6,7,8]. In addition to 5α -reductases, also one 17β -hydrogenase isotype enzyme, namely Aldo-Keto-Reductase 1C3 (full name type 5 17β-hydroxysteroid dehydrogenase (HSD)/prostaglandin (PG) F2α synthase, abbreviated AKR1C3) is implicated in the production of androgens in castration-resistant PCa [9].

#### 1.1.3. 11-Oxygenated Androgens

In connection with adrenal androgens and their role in castration-resistant PCa, until recently, overlooked 11-oxygenated androgens, namely 11β-hydroxyandrostenedione, 11-ketoandrostenedione, 11β-hydroxytestosterone, and 11β-hydroxydihydrotestosterone should be mentioned here. Studies from various research teams brought evidence that 11β-hydroxyandrostenedione, an abundant DHEA metabolite of exclusively adrenal origin (see [10]), serves as the precursor to these latter potent androgens. Their biosynthesis and clinical significance under various clinical conditions were described and reviewed recently [11,12,13].

Using cancer cell lines LNCaP and VCaP, it was proven that they bind to the androgen receptor with affinities close to that of testosterone and dihydrotestosterone and induced both the expression of representative androgen receptor-regulated genes as well as cellular proliferation in the androgen-dependent prostate [14]. Of interest is that these metabolites are less readily deactivated that classical androgens testosterone and DHT and thus remain active longer [11]. Their determination in the prostate tissue may serve as a promising additional marker for PCa development.

#### 1.1.4. Intraprostatic Androgens—A Summary

The biological activity of sex and other steroids in the prostate is influenced by various factors, such as transport mechanisms regulating steroid uptake [15], steroid receptor content, and, last but not least intraprostatic metabolism [7,8,16,17] and environmental factors [18,19]. Consequently, circulating steroid levels may not fully reflect the situation in the prostate, and the measurement of intraprostatic steroid levels is preferred [20]. DHT, T, and also their adrenal androgen precursors are the first at stake. Alternative pathways of androgen biosynthesis from adrenal precursors, including the backdoor pathway and important intermediate products, should be taken into account as well [21].

### 1.2. The Testis

#### 1.2.1. Intratesticular Androgens and Their Forms

The testis is the main organ responsible for spermatogenesis and sperm maturation. About 90% of the T circulating in men originates from Leydig cells. Leydig and Sertoli cells are both involved in the formation of testicular tissue. The differentiation of Leydig cells, which is responsible for T formation, is based on the interplay between Sertoli cell ligands and Leydig cell receptors [22]. The importance of such communication between testicular cells is further stressed by the existence of intratesticular androgen-binding protein (ABP), secreted by Sertoli cells, which mediates intratesticular transport of active androgens between Sertoli and Leydig cells. ABP is the product of the same gene of plasmatic sex hormone-binding globulin (SHBG) [23,24].

Steroid hormones occur in several forms. As intratesticular androgens concerns, we distinguish free, albumin-bound, androgen-binding protein (ABP)-bound and, since the processed tissue is vascularized, it is also bound to SHBG from the circulation. In addition, there are polar conjugates, mainly sulfates, and glucuronides. The preparation of the sample plays an important role as the organic extraction solvents must completely disrupt the binding of the steroid to the protein.

Not only free steroid hormones but also albumin-bound forms are available to the target tissues for biological activity. Therefore, by bioavailable steroid hormone is meant the sum of free and albumin-bound steroids. Its determination is more relevant than its total concentration (the sum of conjugated and unconjugated steroid hormones). The non-ABP/SHBG-bound steroids can be analyzed using ammonium sulfate saturated solution to precipitate ABP/SHBG together with the steroids bound to it, followed by its separation using centrifugation. Moreover, androgen conjugates with glucuronic or sulfuric acid occur in testes as well.

#### 1.2.2. Regulation of Testicular Steroidogenesis

Testicular steroidogenesis undergoes sophisticated regulation, including both non-genomic and genomic signaling. Although luteinizing hormone (LH) is the main hormone responsible for T production, there are other factors involved that affect LH-induced signaling [22]. The testes are not only sites of sex steroid biosynthesis but also target organs containing androgen receptors [24,25] and the site of further steroid metabolism [3,26,27]. Thus, from the point of view of endocrine/paracrine regulation, the testis represents a microenvironment characterized by its own hormone metabolism and regulatory mechanisms; in this microenvironment, the actual steroid concentration is of crucial importance and may differ considerably from circulating steroid hormone levels [24,28].

### 1.3. Conclusive Remarks

Since the first reports into the determination of steroids in testicular and prostatic tissues, methods have advanced considerably with respect to both matrix processing and end-point measurement. However, it is clear from the literature that tissue is sometimes mishandled, particularly prostate tissue, and/or that the most precise measurement method is not always used. This means that a review of the methods used for the analysis of testicular and prostatic steroids is much needed.

Here, we describe and critically evaluate these methods with particular reference to sample collection and processing, analyte separation and final determination, and the clinical informative value of the analyzed intraprostatic, and intratesticular steroids. Although the reasons for the measurement of intra-tissue sex steroids in the prostate and testis differ, the methodologies have much in common. Therefore, we attempted to provide a survey and evaluation of methodical approaches used for sex steroid determination in both tissues.

## 2. Sample Collection and Pre-Analytical Processing Techniques

### 2.1. Prostate

Before the introduction of more sensitive methods for steroid determination, roughly until 2010, slices from surgical removal of the gland by total prostatectomy or samples from core biopsy, either peripheral or periurethral, were the most frequently used materials, which were later replaced by needle biopsy. Depending on the condition and treatment approach, prostatectomy can be performed in several ways. In the case of PCa, radical prostatectomy is usually the method of choice. The entire prostate gland as well as surrounding lymph nodes are removed either by laparoscopic, open, or robot-assisted radical prostatectomy. Simple prostatectomy or transurethral resection of the prostate (TURP) is the procedure to remove the inside part of the prostate gland to treat benign prostate hyperplasia (BPH).

The preanalytical techniques and sample processing methods are summarized in Table 1. Prior to homogenization in water, saline, or a buffer, the tissues are usually cut into pieces or pulverized in liquid nitrogen, and the unconjugated steroids are extracted with organic solvents. β-glucuronidase is typically used for enzymatic hydrolysis to obtain free steroids [29]. The table shows how the various procedures used differ in terms of homogenization techniques and types of extraction. Pre-separation by liquid–liquid or solid phase extraction (SPE) was used. For the liquid–liquid chromatography, diethylether, methyl tert-butyl ether, or acetonitrile are usually used. The SPE can be carried out either using commercially available SPE cartridges or using in-house prepared micro-SPE columns in pipette tips [30]. As a stationary phase, C18 and porous polymer Oasis^®^ HLB phases are the most commonly used ones. The disadvantage of the latter is a high volume of water-containing solvent in the eluate, requiring time-consuming evaporation.

The lower limit of detection (LOD) of the recent methods for the determination of intraprostatic androgens based on the use of needle biopsy published since 2011 amounts to less than 0.5 and 1.0 pg/sample for T and DHT, respectively [15,20,31,32]. It enables simple extraction of the homogenized tissue, and so, such methods are to be recommended for further research. As may be seen in Table 1, in most methods, the pre-separation of steroids by extraction (organic solvents, solvent partition, solid-phase extraction) before a final determination was sufficient, only in one instance, the pre-separation of steroids by HPLC was used as well [33].

The choice of a type of tissue depends on many factors such as the size and the type of tissue, surgeons’ approach, but immediate freezing of the obtained tissue is a necessary step to maintain actual concentrations. As was already mentioned, the concentration of DHT decreases by more than half within 2 h of incubation at 37 °C, indicating the further metabolism of DHT. Cutting or mincing followed by homogenization of the tissue should precede extraction of the steroid hormones. The choice of non-polar solvents is up to the laboratory, but the use of stable isotope internal standards is highly recommended, as it corrects the matrix effect and possible analyte loss during the extraction procedure. Deconjugation of polar conjugates should precede the measurement of the total steroid hormones concentration (the sum of conjugated and unconjugated forms).

### 2.2. Testes

As shown in Table 2, most authors used for steroid analyses intratesticular fluid obtained by percutaneous testicular aspiration (PTA) or fine needle aspiration (FNA), the tissue by orchiectomy, or microsurgical testicular sperm extraction (M-TESE) [24,42,43,44]. PTA is an easy and cheap method of sperm collection performed by sticking a needle (in case of FNA, a fine needle) and aspirating testicular fluid with negative pressure. With M-TESE, sperm is collected directly from the testicular tissue using an operating microscope, which allows the surgeon to find regions of seminiferous tubules of the testes that are more likely to contain spermatozoa.

In the case of intratesticular fluid, only extraction and separation of the analytes is necessary, while in the tissue, grinding in buffer using a suitable homogenizer (e.g., ultra-turrax) or mechanical pulverizing of the frozen tissue in liquid nitrogen were usually used. Next, various organic solvents were used to extract the steroids, once followed by partitioning the water and organic solvent in order to separate the polar compounds [45]. In some cases, additional chromatographical separation steps, such as SPE high-performance liquid chromatography (HPLC), and Celite column partition chromatography are performed. After evaporation, the samples are subjected to final steroid determination, as in the case of the prostate. T is the main intra-tissue androgen in the testis, the concentration of which exceeds that of DHT by almost two orders of magnitude (see Table 3. Therefore, pre-separation of steroids by HPLC had to be involved, regardless of whether immunoassay or liquid chromatography-mass spectrometry (LC-MS) were used for final determination. Especially when using immunoanalytical methods (IA), the separation of cross-reacting compounds is essential. The studies using intratesticular fluid listed in Table 2 date back before 2010, reflecting the methods available of that time and, moreover, some of them are from the same author’s group. Therefore, it is difficult at present to recommend the best methodical approach, and new studies are needed to take advantage of recent methodology.

## 3. Determination of Steroid Hormones

To determine conjugated steroid hormones, the addition of ammonium sulfate and acidification to pH 2 with 10% HCl prior to extraction with diethylether:isopropanol mixture (3:1, *v*/*v*) enables the extraction of these conjugates to polar organic solvent [52]. When determining the total amount of the steroid (conjugated and unconjugated forms), disruption of the binding to the proteins and deconjugation of the polar conjugates must be performed. Enzymatic hydrolysis using β-glucuronidase to deconjugation is a common approach. It is necessary to consider that enzyme kinetics vary depending on the steroid substrate, pH, and temperature. The use of deconjugation standards such as 4-methylumbelliferyl sulfate or glucuronide is highly recommended.

Currently, the most widely used methods to determine steroid hormone levels are IA methods and chromatographic techniques coupled with MS. Both approaches possess advantages and disadvantages.

### 3.1. Immunoanalytical Methods

Immunoassay-based methods are fast and relatively easy to perform, with no requirement for highly qualified personnel—which, together with lower instrumentation costs, means that these methods are more widespread in clinical practice. They are based on the detection of the immunocomplex that arises when an antibody interacts with a target analyte. Radioimmunoassay (RIA) uses a radionuclide as an indicator and detects radioactivity on a gamma counter, whereas chemiluminescence immunoassay (CLIA) uses the transformation of chemical energy into light energy. The quality of results may be affected by cross-reactivity between similar substances, which can lead to inaccuracies and cause low method specificity and sensitivity. Another disadvantage of immunoassays consists of the fact that the antisera are constructed for a single steroid, and thus, these methods cannot be used to quantify arrays of steroids as is possible with LC-MS/MS and GC-MS/MS. This problem can be partially solved by combining IA-based methods with liquid or gas chromatography (LC, GC) for prior analyte separation. The disadvantage of this combined approach to steroid hormone determination is that it increases operating costs and that skilled operators are required for such analysis.

### 3.2. Mass Spectrometry Methods

The determination of steroid hormone levels can also be carried out solely using GC- or LC-based methods coupled with MS detection, in which ionized molecules are separated according to their mass-to-charge ratio.

Due to the low volatility of steroids, derivatization is an essential part of the analyses performed using GC-MS. In general, silylation under strictly anhydrous conditions is the most relevant approach in the analysis of steroids having hydroxyls in their structure. Trimethylsilyl is the most frequently used one among various alkyl silyl groups [53]. Phenolic hydroxyls are typically treated under the same conditions as hydroxyls. Another approach is acylation, which is mostly performed with acid anhydrides or acyl halides. Except for hydroxyls, carbonyl groups are a common target of the derivatization. Methoxyamine, hydroxylamine, and *N, N*-dimethylhydrazine are the most widespread. Due to possible keto-enolic tautomerism, carbonyl groups can also undergo sylilation with *N*-methyl-*N*-trimethylsilyltrifluoractamide, which is used for the simultaneous derivatization of hydroxyls and carbonyl [54,55,56].

In LC-MS analysis, derivatization is nowadays not a necessity but often used to enhance the signal intensity. Conversion of the hydroxyl to an ester by the mixed anhydride method with the use of picolinic or fusaric acid was firstly described by Yamashita. Such a procedure finds its application in the analysis of estrogens but also androgens and corticosteroids [57,58,59]. T and DHT derivatized as picolinyl esters were determined in both human serum and prostate, as well as in rat testicular fluid together with estradiol (E2) and 3α,5α-androstanediol [37,60]. O’Brien et al. developed a derivatization method for the determination of androgens with hydroxy group utilizing 2-fluoro-1-methylpyridinium *p*-toluenesulfonate, which was later used for the determination of T, DHT, and all isomers of androstanediols in the prostate [61,62]. For the derivatization of the keto group of androgens and several corticosteroids, hydrazine-based reagents are often used. 2-hydrazino-1-methylpyridine, Girard reagent T, and 2-hydrazino-4-trifluoromethylpyrimidine are among the most widespread [63,64,65,66]. The last mentioned, 2-hydrazino-4-trifluoromethylpyrimidine, was used for the analysis of T and DHT in various mouse tissues, such as testis, seminal vesicle, and prostate [67]. For a detailed review on possible steroid derivatization approaches in LC-MS, see the review published by Higashi and Ogawa [68].

In both methodologies, stable isotope internal standards are used during the extraction and measurement, as it diminishes the risk for quantification of wrong analytes and corrects the possible analyte loss during the extraction procedure [24,28].

As a mobile phase, an inert gas (usually helium) is used in gas chromatography, while the mixture of polar solvents (most commonly methanol, water, and acetonitrile) which can be enriched with the addition of formic acid, ammonium formate, ammonium fluoride, or some other additive enhancing the ionization of analytes, is used in liquid chromatography [69]. The correct choice of a mobile phase gradient is often crucial in the separation of the analytes. C18 reverse-phase is among the mostly used in the analysis of steroid hormones. For the analysis of compounds with an aromatic ring in its structure, phenyl, biphenyl, or phenyl-hexyl phases are also possible for the application because of their π–π interaction.

The most commonly used analyzers are triple quadrupole, mostly operating in multiple reaction mode, and ion trap. Devices combining triple quadrupole and ion trap and thus connecting both techniques are already available and are currently used in small molecule analysis. LC-MS with an electrospray ionization interface has become the most widespread choice for steroid analysis. As it offers an opportunity of choice between positive and negative ionization mode, both are used in the steroid analysis. A positive ionization mode is the most often used, but for the analysis of estrogens, and aldosterone, negative ionization mode is often a better choice. The use of ammonium fluoride as a mobile phase additive to support ionization in the negative mode is highly recommended.

Such methods are particularly applicable to the analysis of substances of very low concentrations, as well as when a simultaneous determination of a larger number of analytes is necessary. When using the correctly chosen method, many analytes can be quantified simultaneously in a short time with high sensitivity, specificity, and accuracy. MS analysis requires more expensive instruments and highly skilled technicians, meaning that these methods are to this date not the most widespread in routine clinical practice but mainly used in research. However, for those substances for which there are no commercially available IA kits, LC-MS and GC-MS methods become indispensable.

The sensitivity of IA methods for the determination of intraprostatic DHT and T is usually not sufficient, and the levels of steroid hormones are often below LOD. A greater amount of the tissue (usually hundreds of mg) is usually needed. On the other hand, modern chromatographic techniques coupled with MS reached the LOD of the above androgens 0.02 pg/mg tissue, enabling taking into analysis one tissue sample obtained by needle biopsy.

The methods used for end-point steroid determination, including eventual derivatization, in the prostate are summarized in Table 1 and those for the testis are summarized in Table 2.

## 4. Clinical Information Value of Intraprostatic Steroid Concentrations for Diagnosis of Prostate Cancer

Major questions concerning the diagnostic value of steroid determination in the blood or in prostatic tissue are as follows: (i) How do the levels of steroid hormones help distinguish BPH from PCa? (ii) How do these levels help us predict PCa development? 

The concentrations of the most important androgens, as measured in both prostatic tissue and blood, are shown in Table 4. There is a general consensus that there is little or no correlation between, on the one hand, intraprostatic T and DHT concentrations and, on the other, blood levels; this finding is consistent, regardless of whether the subject has PCa or not [20,33,34,38]. Though application of transdermal DHT to healthy men led to increased serum DHT (associated with decreased serum T), it did not significantly alter the intraprostatic levels of either androgen [26]. Similarly, in a study in which otherwise-healthy medically castrated men received exogenous T, the administration resulted in dose-dependent increases in serum T and DHT concentrations, while the intraprostatic androgen concentration (predominantly DHT) remained stable across the physiological range [35].

Although the consensus is that intraprostatic steroid determination is more reliable than determining steroid levels in the blood, its diagnostic value is not unequivocal. This is since in most studies, intraprostatic steroid concentration is influenced by many factors.

### 4.1. Subject Group

The subject group often includes at least two of the following subgroups (see Table 1): subjects suspected from BPH or PCa and scheduled for biopsy; patients with a confirmed diagnosis; those who have already undergone surgery for BPH or PCa.

A large study of 196 men with PCa [34] found high T levels in prostate tissue, which were related to a high Gleason score, an advanced clinical stage, and a high percentage of positive biopsy cores. In this study, tissue was obtained by radical prostatectomy and biopsy followed by LC-MS/MS analysis of derivatized steroid hormones. The authors concluded that T level in a needle biopsy specimen may be a useful prognostic factor in PCa. Similar results were obtained in an earlier study of surgical samples obtained from simple and radical prostatectomy, where the analysis was conducted by HPLC-RIA [33]. However, in a recent study [36] of 93 patients scheduled for initial prostate biopsy, no significant differences were found in T and DHT tissue levels between patients with BPH and with PCa. An analysis of T and DHT was performed using LC-MS/MS, and, surprisingly, another study [31] reported that PCa patients with a high Gleason score (7 to 10) had relatively low intraprostatic DHT concentrations (analyzed by LC-MS/MS), which were still sufficient to activate androgen receptor expression and propagate tumor growth.

### 4.2. Extragonadal Androgen Sources

Concerning castration-resistant PCa (CRPC), of particular importance are products of intraprostatic metabolites of adrenal androgens, the synthesis of which is independent of the gonadal–hypothalamic pituitary axis. As many as five biosynthetic pathways may take place by which adrenal steroids are converted to the potent male sex hormones, testosterone, and dihydrotestosterone [21,70,71]. Moreover, already mentioned 11-oxygenated androgens, intraprostatic metabolites of 11β-hydroxyandrostenedione, should be taken into consideration.

### 4.3. Technique and Site (Core) of Sample Removal

A perspective approach is the collection of samples from benign as well as the malignant part of the tissue as checked histologically (see Sample collection and processing techniques) [34].

### 4.4. Choice of End-Point Steroid Determination

Advantages and disadvantages of the currently most widely used methods for the determination of steroid hormones (IA and LC-MS or GC-MS) were already discussed in Section 3. Determination of steroid hormones.

### 4.5. Applied Treatment

A serious problem is the recurrence of PCa even after ADT. One study compared the intraprostatic levels of T and DHT in the cancer specimens of 18 men whose cancer returned during ADT and in the benign prostate specimens of 18 men receiving hormonal treatment [39]. The authors found similar T concentrations in recurrent PCa and androgen-stimulated benign prostate, while DHT levels decreased by 91% in the recurrent PCa prostate. The concentrations of steroid hormones were analyzed by LC-MS/MS. They concluded that recurrent PCa prostate may develop the capacity to biosynthesize testicular androgens from adrenal androgens or cholesterol. These results are in agreement with the earlier study of Mostaghel et al. [32], who showed that no form of ADT can completely eliminate intraprostatic androgens, which relates to their extragonadal origin. Mostaghel et al. used both GC-MS/MS and IA methods.

### 4.6. The Role of Intraprostatic Metabolism

Metabolic transformations of steroids were studied as early as in the seventies, see e.g., [72,73]; for a review, see [74]. Neuzillet et al. [40] recently investigated the activity of steroid metabolizing enzymes and their in situ expression. Men not diagnosed with PCa were included in their STERPROSER (urological) study of the relationship between, on the one hand, intraprostatic androgens, major estrogens, and androgen precursors, and on the other, prostate volume. These authors reported higher DHT concentrations measured by GC-MS/MS in high-volume prostates, which they attributed to either higher 5α-reductase expression or the lower expression of downstream metabolizing enzymes.

In another study, Olsson et al. [38] showed that intraprostatic DHT concentration depends on its inactivation (metabolism) to isomeric adiol-glucuronides by uridine diphospho (UDP)-glucuronosyl transferase (UGT) enzymes. They demonstrated an association between the DHT concentrations and genetic mutations of these enzymes. GC-MS/MS and IA methods were used for the determination of androgens. Similarly, Lévesque et al. [29] found an association between intraprostatic androgen concentrations in PCa patients and polymorphisms in genes encoding androgen 5α-reductase (SRD5A1, SRD5A2). Androgens in this study were measured by GC-MS/MS. Moreover, Mostaghel et al. [15] investigated whether genetic variations in SLCO genes may serve as predictors of PCa patient response to abiraterone treatment. They found that the levels of both intraprostatic abiraterone and intraprostatic androgen are associated with genetic variations in SLCO2B1. Since abiraterone, a commonly used 17α-hydroxylase, 17,20-lyase (CYP17A1) inhibitor, is transported to the prostate via (ubiquitous) SLCO, this raises the possibility of the successful chemical treatment of PCa by the targeted drug delivery. LC-MS/MS analysis was performed in this study.

### 4.7. Other Factors

Recently, researchers have investigated the possible association between male pattern baldness and serum and intraprostatic androgen levels [41]. In a large study of 248 PCa patients, the authors found a strong correlation between baldness and serum T and DHT, but only a weak association with elevated intraprostatic T. RIA was used in this study in order to analyze steroid hormone concentration. Conversely, neither circulating nor intraprostatic sex hormones were significantly associated with chest hair density. These findings point to differences between the androgen receptor polymorphism and the roles they play [75].

## 5. Relationship between Intratesticular and Blood Steroid Concentrations: Clinical Information Value for Diagnosis

In contrast to intraprostatic steroids, far fewer papers have focused on the determination of intratesticular steroids. Such papers have investigated the relationship between intratesticular steroid concentrations and serum levels in order to determine how these levels reflect spermatogenesis in healthy men and men with impaired sperm function. In United States, two major research groups have addressed this issue: the Baltimore group [24,28,42] and the Seattle group [44,46,47,48,76]. In all of their studies, the authors used intratesticular fluid obtained using various types of needle biopsy. In Finland, Huhtaniemi’s group focused on the determination of steroid hormones and steroid hormone sulfates from testicular tissue already in the eighties [50,77] (Table 3).

While their data differ somewhat, all of the groups show that in fertile men, steroid concentrations in the intratesticular fluid are up to two orders of magnitude higher than their corresponding serum levels [24,28,42,44,46,47,48,76]. Similar levels were found in the testicular biopsy tissue of 84 azoospermic men using a combination of HPLC separation and RIA [45]. In contrast to plasma, the concentrations of SHBG/ABP in the testicular fluid were so low that most T was in the free form [24]. Studies of Jarow’s group [24,28] were conducted on the same patient cohort. Generally, intratesticular DHT concentrations are much lower than T levels and similar to those found in serum, thereby confirming that T in the testis is the principal androgen. [42,44,45,48].

Some of these studies have reported controversial results regarding the relationship between intratesticular and serum T concentrations. In a study of 22 healthy men, T concentrations in the serum measured by LC-MS/MS did not correlate with levels in the intratesticular fluid [42]. In fact, the mean intratesticular T concentration was similar to that previously obtained by RIA [24]. Moreover, no correlation was found between sperm count and total intratesticular T concentration. Our study of 85 azoospermic men also showed poor or no correlation between T and DHT in the testicular tissue and in the serum [45]. The main aim of some later studies was to investigate the impact of a male hormonal contraceptive regime (peroral treatment with a combination of tenanthate and levonorgestrel) on intratesticular T concentration [46,47]. Functioning spermatogenesis requires a certain T concentration within the testis, which, under physiological conditions, amounts to a hundred times higher concentration that in the serum; when intratesticular T levels were suppressed by 98%, to a level comparable with serum level, it was insufficient to support normal spermatogenesis. In another Seattle study, the dose-dependent effect of human chorionic gonadotropin (hCG) on intratesticular T levels was investigated in relation to the hormonal regulation of spermatogenesis [76]. These researchers administered increasing doses of hCG to 37 normal men in whom experimental gonadotropin deficiency had been induced by the GnRH antagonist acyline. Even low doses of hCG were sufficient to reach the intratesticular T level needed for normal spermatogenesis.

In all these studies, only a small number of subjects were investigated and, thus, it is evident that more studies are needed on the relationship between intratesticular and circulating steroid concentrations.

## 6. Conclusions

The diagnosis of PCa, BPH, and male fertility disorders involves the analysis of main androgens. Since blood serum does not always reflect the actual situation in affected tissues, as repeatedly evidenced by poor correlation between intratissue and blood steroid levels, the determination in the tissues is to be preferred. For this reason, we have summarized the state of the art in using the intratissue concentrations of the main sex steroid players for diagnostic purposes. 

We have shown that the methodical approaches chosen at each stage (material collection, preanalytical processing, final steroid determination) of the analysis of intraprostatic and intratesticular steroids is critical.

The following conclusions may be drawn from the reviewed data and presented tables:

1. Advanced analytical tools, such as LC- or GC-MS/MS, best facilitate the analysis of small amounts of prostatic tissue from needle biopsy, as well as providing a more patient-friendly approach.
2. DHT is the main intraprostatic androgen, its concentration being about ten times that of blood serum.
3. Although intraprostatic DHT is higher in patients with PCa than in other groups in most instances, available data from various researcher groups are not sufficient to distinguish definitely patients with PCa from other subjects. The results strongly depend on methodology, namely analyzed material, sample collection, separation techniques, and end-point measurement. Moreover, there is very little information from healthy men. Only results obtained by the same or similar methodology can be compared seriously.
4. T is the main intratesticular androgen, its concentration exceeding blood levels by almost two orders of magnitude, but very little information is available from men with fertility disorders.
5. There is no consensus as to whether intratesticular T concentrations correlate with serum levels.
6. Intratesticular androgens respond either poorly or not at all to exogenous hormone administration.
7. 11-oxygenated androgens as dominant active androgens are promising biomarkers in the evaluation and diagnosis of androgen-dependent diseases. Of crucial importance is their contribution to CRPC progression.

Taken together, these conclusions show that more data are needed on the intraprostatic androgens. Such data are required for both BPH and PCa patients and, in the latter case, they should factor in disease severity. Crucially, such data need to be obtained using a standardized methodology, such as needle biopsy with LC-MS/MS. Furthermore, greater focus on determining the levels of a wider range of intratesticular steroid metabolites, such as those of adrenal origin, would enhance our understanding of other biosynthetic pathways and thereby could contribute to the improved diagnosis of PCa and male fertility disorders.

## Figures and Tables

**Table 1:** Survey of techniques used for intraprostatic steroid determination.

Probands	Diagnosis	Material	Technique of Tissue Processing	Determination	Intraprostatic Steroids	Serum Steroid and Other Hormones	Reference
196	PCa	B, RP	cutting, homogenization, protein precipitation, SPE extraction, derivatization	LC-MS/MS	DHT, T	DHT, T	[34]
27	H	B	homogenization, extraction, derivatization	LC-MS/MS, RIA	DHT, T, adione, DHEA	DHT, T, adione, E2, DHEA, LH, FSH, SHBG	[26]
51	H	B	extraction, derivatization	LC-MS/MS	DHT, T, adione, DHEA, preg, prog, 17α-OH-preg,17α-OH-prog	DHT, T	[35]
58	PCa	B, RP	tissue heating to 60 °C, homogenization, extraction, derivatization	LC-MS/MS	DHT, T, adione, DHEA, preg, prog	DHT, T, adione, DHEA, preg, prog	[15]
526	PCa	RP	homogenization, extraction, enzymatic deconjugation, derivatization, SPE extraction	GC-MS/MS	DHT, T, DHEA, 5diol, 3β-diol, androsterone	DHT, T, adione, E2, DHEA, DHEA-S, 5diol, 3β-diol, ADT, E1, E1-S, ADT-G, 3α-diol-3G, 3α-diol-17G	[29]
251	PCa	RP	cutting, mincing, extraction, celite column partition chromatography	celite column partition-RIA	DHT, T, adione, E2, E1, 3α-diol G	DHT, T, adione, E2, E1, 3α-diol-G	[20]
93	BPH (48), PCa (45)	B	chopping, extraction (in an ice-cooling ultrasonic bath), extraction, SPE extraction	LC-MS/MS	DHT, T	none	[36]
67	BPH (30), PCa (37)	B	homogenization in an ice-cooling bath, protein precipitation with EtOH, SPE extraction, derivatization	LC-MS/MS	DHT, T	DHT, T	[37]
178	BPH (57), PCa (121)	SP/RP	homogenization, double extraction, HPLC column separation	HPLC-RIA	DHT, T, adione, epiT	DHT, T, SHBG	[33]
16	PCa	RP, peripheral serum, serum from the prostatic veins	homogenization, precipitation, extraction, derivatization	GC-MS/MS, IA	DHT, T, androsterone, 3α-diol-3G, 3α-diol-17G, ADT-G	DHT, T, SHBG, LH, FSH	[38]
36	PCa with ADT (18), AS-BP (18)	RP, TURP	homogenization at 4 °C, extraction, SPE extraction	LC-MS/MS	DHT, T	none	[39]
81	PCa (47), H (34)	B	dissolution of DHT in alkalite solution, SPE extraction, derivatization	LC-MS/MS (DHT), RIA	DHT	T, adione, DHEA, DHEA-S, ACTH, F, PRL, LH, FSH	[31]
35	PCa	RP	homogenization, extraction, derivatization	GC-MS/MS, IA	DHT, T, androsterone, 3α-diol-3G, 3α-diol-17G, ADT-G	DHT, T, SHBG, LH, FSH	[32]
32	BPH, bladder cancer	SP, RC	frozen tissue pulverizing, homogenization at 4 °C, SPE extraction, solvolysis of conjugates with sulfuric acid, derivatization	GC-MS/MS, RIA	DHT, T, E2, DHEA, DHEA-S, E1,5-diol	DHT, T, E2, DHEA, DHEA-S, E1, 5-diol, FSH, LH, SHBG	[40]
248	PCa	RP	homogenization, extraction	RIA	DHT, T, adione, E2, 3α-diol-G, E1,	DHT, T, adione, E2, E1, 3α-diol-G, SHBG	[41]
3	BPH	TURP	lyophilization, extraction, enzymatic deconjugation	UHPSFC-MS	DHT, T, adione, 3α-diol, other androgen metabolites		[11]

Abbreviations: 17α-OH-preg, 17α-hydroxypregnenolone; 17α-OH-prog, 17α-hydroxyprogesterone; 3α-diol, 5α-androstane-3α,17β-diol; 3α-diol-17G, 3α-diol-17-glucuronide; 3α-diol-G, 3α-diol-3-glucuronide; 3β-diol, androstane-3β-17β-diol; 5-diol, 5-androsten-3β, 17β-diol; ABP, androgen-binding protein; adiol, androstenediol; adione, androstenedione; ADT, androgen deprivation therapy; ADT-G, androsterone-glucuronide; AS-BP, androgen stimulated benign prostate; B, biopsy; BP, benign prostate; BPH, benign prostatic hyperplasia; DHEA, dehydroepiandrosterone; DHEA-S, dehydroepiandrosterone-sulfate; DHT, 5α-dihydrotestosterone; E1, estrone; E2, estradiol; EpiT, epitestosterone; EtOH, ethanol; FNA, fine needle percutaneous testicular aspiration; FSH, follicle-stimulating hormone; GC-MS/MS, gas chromatography tandem mass spectrometry; H, healthy; HPLC, high-performance liquid chromatography; hCG, human chorionic gonadotropin; IA, immunoanalytical; LC-MS/MS, liquid chromatography tandem mass spectrometry; LH, luteinizing hormone; PCa, prostate cancer; preg, pregnenolone; PRL, prolactin; prog, progesterone; PTA, percutaneous testicular aspiration; RC, radical cystectomy; RP, radical prostatectomy; SLCO, solute carrier organic anion protein; SHBG, sex hormone-binding globulin; SP, simple prostatectomy; SPE, solid phase extraction; T, testosterone; TURP, transurethral resection of the prostate; UHPSFC-MS, ultra-high performance supercritical fluid chromatography tandem mass spectrometry.

**Table 2:** Survey of techniques used for intratesticular steroid determination.

Probands	Diagnosis	Material	Technique of Material Processing	Determination	Intratesticular Hormones	Serum Steroids and Other Hormones	Reference
22	H, undergoing vasectomy	intratesticular fluid (PTA)	centrifugation, extraction, HPLC separation	RIA	DHT, T, SHBG/ABP	T	[24]
84	azoospermic	testicular tissue (M-TESE)	homogenization, extraction, solvent partition, HPLC separation	HPLC-RIA	DHT, T, adione, E2, EpiT	T, E2, LH, FSH, PRL, SHBG	[45]
10	H	intratesticular fluid (PTA)	centriguation, extraction, derivatization	LC-MS/MS	DHT, T, E2	T, DHT, E2	[44]
10	H	intratesticular fluid (PTA)	centrifugation, extraction, HPLC separation	LC-MS/MS	DHT, T, E2, 3α-diol	not given	[42]
7	H	intratesticular fluid (PTA)	centrifugation, extraction	IA	T	LH, FSH, T	[46]
29	H	intratesticular fluid (FNA)	centrifugation, extraction	RIA	T	LH, FSH, T	[47]
20	H	intratesticular fluid (FNA)	centrifugation, extraction	LC-MS/MS	DHT, T	T, LH, FSH	[48]
52	old men with PCa/young men with varicocele	orchiectomy of prostatic carcinoma/biopsy of varicocele testes	homogenization, centrifugation, extraction, Sephadex column chromatography	RIA	T	T, LH, FSH	[49]
17	PCa	orchiectomy	homogenization, extraction, column chromatography separation	RIA	DHT, T, adione	T, LH, FSH, SHBG	[50,51]

Abbreviations: 3α-diol, 5α-androstane-3α,17β-diol; ABP, androgen-binding protein; adiol, androstenediol; adione, androstenedione; ADT-G, androsterone-glucuronide; DHT, 5α-dihydrotestosterone; E2, estradiol; EpiT, epitestosterone; FNA, fine needle percutaneous testicular aspiration; FSH, follicle-stimulating hormone; H, healthy; HPLC, high-performance liquid chromatography; IA, immunoanalytical; LC-MS/MS, liquid chromatography tandem mass spectrometry; LH, luteinizing hormone; M-TESE, microsurgical testicular sperm extraction; PCa, prostate cancer; PRL, prolactin; PTA, percutaneous testicular aspiration; SHBG, sex hormone-binding globulin; T, testosterone.

**Table 3:** Levels of major intratesticular and serum steroid hormones.

Diagnosis	Concentrations Testes [ng/mL]	Concentrations Serum [ng/mL]	Reference
H, undergoing vasectomy	DHT [not detectable], T [356 ± 24.8], ×	T [3.7 ± 0.3], ×	[24]
azoospermic	DHT [98.8 ± 145], T [603 ± 684], adione [22.9 ± 37.2], E2 [19,067 ± 24,514], £	T [0.004 ± 0.001], E2 [0.021 ± 0.009], £	[45]
H	DHT [3.7,(1.1–4.7)], T [486 (429–897)], E2 [2.7,(1.3–2.4)], §	DHT [0.2,(0.12–0.24)], T [3.0,(2.3–3.9)], E2 [(0.025,(0.019–0.029)], §	[44]
H	DHT [13.4 ± 1.8], T [572 ± 102], E2 [15.7 ± 2.3], £	none	[42]
H after hormonal contraception	T [238 ± 39.3], ×	T [6.59 ± 0.55], ×	[46]
H after hormonal contraception	T [339 ± 22.83], ×	T [4.07 ± 0.32], ×	[47]
H after male contraceptive treatment	DHT [1.48 ± 0.23], T [10.12 ± 2.31], £	none	[48]
old men with PCa/young men with varicocele	aged men T [860 ± 530 ng/g tissue] × young men T [1700 ± 1100 ng/g tissue] ×	aged men T [3.34 ± 1.93] × young men T [6.42 ± 1.91] ×	[49]
PCa	T (not treated) [398 ± 60 ng/g tissue] ×	dependent on the treatment	[50,51]

Values presented as: ¥ median with 95% confidence interval (CI), § medians with interquartile ranges, × geometric mean ± standard error of the mean (SEM), £ median ± standard deviation (SD); Abbreviations: adione, androstenedione; DHT, 5α-dihydrotestosterone; E2, estradiol; H, healthy; PCa, prostate cancer; T, testosterone.

**Table 4:** Levels of major intraprostatic and serum steroid hormones.

Diagnosis	Concentrations Tissue [ng/g]	Concentrations Serum [ng/mL]	Reference
PCa	DHT [7.06, (8.75–11.63)], T [0.57, (0.94–1.38)], ¥	DHT [0.33 (0.33–0.38)], T [(3.55 [3.49–3.90)] ¥	[34]
H	DHT [2.8 ± 0.2], T [0.6 ± 0.2], adione [0.27 ± 0.03], ×	adione [0.8 ± 0.2], ×	[26]
H	DHT [4.03], T [0.22], adione [0.16, (1, 0.33)], §	DHT [0.36, (0.27, 0.47)], T [4.50, (3.15, 5.16)], §	[35]
PCa	DHT [2.08 ± 0.17], T [0.67 ± 0.16]	DHT and T not given	[29]
PCa	DHT [6.81 (5.3, 8.06), T [0.215, (0.17, 0.29)], adione [0.58 (0.42, 0.82)], E2 [0.05 (0.04, 0.07)], §	DHT [(0.50 (0.38, 0.67)], T [4.68 (3.4, 6.21)], adione [0.69 (0.52, 0.91)], E2 [0.03 (0.02, 0.04)], §	[20]
BPH (48), PCa (45),	DHT [10.3 ± 7.2], T [0.79 ± 0.66], DHT range: [0.98–24.9, median 7.7], T range: [0.1–3.12, median 0.53], £	not given	[36]
BPH (30), PCa (37)	BPH: DHT [7.39 ± 3.08], T [0.37 ± 0.23], PCa: DHT [7.52 ± 3.65], T [0.85 ± 0.92], £	DHT [0.48 ± 0.17], T [4.77 ± 1.53], £	[37]
BPH (57), PCa (121)	BPH: DHT [1.87 ± 1.87], T [0.99 ± 1.31], adione [1.14 ± 1.53], PCa: DHT [2.57 ± 1.97], T [1.32 ± 2.02], adione [1.42 ± 1.28], ×	BPH: DHT [0.62 ± 0.2], T [4.56 ± 2.19], PCa: T [4.25 ± 1.98], DHT [0.59 ± 0.21], ×	[33]
PCa	DHT [5.96 ± 0.61], T [ < 0.0374—under LOD], ×	peripheral: DHT [0.48 ± 0.006], T [3.06 ± 0.2], local: DHT [0.95 ± 0.004], T [2.83 ± 0.18], ×	[38]
PCa with ADT (18), AS-BP (18)	AS-BP: DHT [3.98], T [0.79], PCa: DHT [0.36], T [1.08], ¢	not given	[39]
PCa (47), H (34)	PCa: DHT [5.41 ± 2.55], suspected without PCa: DHT [5.61 ± 1.96], £	PCa: DHT [0.51 ± 0.27], T [4.45], adione [0.81 ± 0.35],H: DHT [0.44 ± 0.23], T [4.25 ± 1.33], adione [0.86 ± 0.41], £	[31]
PCa	DHT [4.38 ± 0.99], T [0.26 ± 0.17], £	range of T in castrate men: [0.094–0.27]	[32]
BPH, bladder cancer	high volume prostate: DHT [6.1, (5.5–6.7)], T [0.43, (0.24–0.61)], E2 [0.0155, (0.0115–0.0194)] normal volume prostate: DHT [5.1, (4.5–5.7)], T [0.47, (0.29–0.66)], E2 [0.02, (0.02–0.03)]	high volume prostate: DHT [0.4, (0.3–0.5)], T [4.4, (3.6–5.3)], E2 [0.03, (0.02–0.03)] normal volume prostate: DHT [0.4, (0.3–0.5)], T [5.0, (4.1–6)], E2 [28.7, (24.3–32.7)]	[40]
PCa	DHT [6.43, (6.08, 6.81)], T [0.21, (0.19, 0.22)], adione [0.59, (0.54, 0.65)], E2 [0.04, (0.04, 0.05)], *	DHT [0.46, (0.43, 0.5)], adione [0.68, (0.63, 0.73)], E2 [0.03, (0.03, 0.03)], *	[41]
BPH	adione [~7.5]		[11]

Values presented as: ¥ median with 95% confidence interval (CI), § medians with interquartile ranges, × geometric mean ± standard error of the mean (SEM), £ median ± standard deviation (SD), ¢ median, * geometric mean with 95% CI. Abbreviations: adione, androstenedione; ADT, androgen deprivation therapy; AS-BP, androgen stimulated benign prostate; BPH, benign prostatic hyperplasia; DHT, 5α-dihydrotestosterone; E2, estradiol; H, healthy; PCa, prostate cancer; T, testosterone.

## Data Availability

Data available in a publicly accessible repository. The data presented in this study are openly available under reference numbers.

## Abbreviations

17α-OH-prog	17α-hydroxyprogesterone; 17-hydroxypregn-4-ene-3,20-dione
3α-diol	5α-androstane-3α,17β-diol
3α-diol-17G	3α-diol-17-glucuronide; 3α-hydroxy-5α-androstan-17β-yl D-glucopyranosiduronic acid
3α-diol-G	3α-diol-3-glucuronide; 3β-hydroxy-5α-androstan-3α-yl D-glucopyranosiduronic acid
3β-diol	androstane-3β,17β-diol
5-diol	5-androsten-3β,17β-diol; androst-5-ene-3β,17β-diol
ABP	androgen binding protein
adiol	androstenediol; androst-5-ene-3β,17β-diol
adione	androstenedione; androst-4-ene-3,17-dione
ADT	androgen deprivation therapy
ADT-G	androsterone glucuronide; 17-oxo-5α-androstan-3α-yl β-D-glucopyranosiduronic acid
AS-BP	androgen stimulated benign prostate
B	biopsy
BP	benign prostate
BPH	benign prostatic hyperplasia
DHEA	dehydroepiandrosterone; 3β-hydroxyandrost-5-en-17-one
DHEA-S	dehydroepiandrosterone-sulfate; 17-oxoandrost-5-en-3β-yl sulfate
DHT	5α-dihydrotestosterone; 17β-hydroxy-5α-androstan-3-one
E1	estrone; 3-hydroxyestra-1(10),2,4-trien-17-one
E2	estradiol; estra-1(10),2,4-triene-3,17β-diol
EpiT	epitestosterone; 17α-hydroxyandrost-4-en-3-one
EtOH	ethanol
FNA	fine needle percutaneous testicular aspiration
FSH	follicle-stimulating hormone
GC-MS/MS	gas chromatography tandem mass spectrometry
H	healthy
HPLC	high-performance liquid chromatography
hCG	human chorionic gonadotropin
IA	immunoanalytical
LC-MS/MS	liquid chromatography tandem mass spectrometry
LH	luteinizing hormone
M-TESE	microsurgical testicular sperm extraction
PCa	prostate cancer
preg	pregnenolone; 3β-hydroxypregn-5-en-20-one
PRL	prolactin
prog	progesterone; pregn-4-ene-3,20-dione
PTA	percutaneous testicular aspiration
RC	radical cystectomy
RP	radical prostatectomy
SLCO	solute carrier organic anion protein
SHBG	sex hormone-binding globulin
SP	simple prostatectomy
SPE	solid phase extraction
T	testosterone; 17β-hydroxyandrost-4-en-3-one
TURP	transurethral resection of the prostate
UHPSFC-MS	ultra-high performance supercritical fluid chromatography tandem mass spectrometry
